# Mycorrhizal fungi arbuscular in organic and conventional sugarcane systems

**DOI:** 10.1038/s41598-024-65358-w

**Published:** 2024-06-21

**Authors:** Jadson Belem de Moura, Maria Lucrécia Gerosa Ramos, Maria Luiza de Freitas Konrad, Orivaldo José Saggin Júnior, Sandro Dutra e Silva

**Affiliations:** 1 Graduate Studies in Social, Technological and Environment Science, Evangelical University of Goiás, Anápolis, Goiás Brazil; 2 Sedmo - Soil Research Group, Ecology and Dynamics of Organic Matter, Evangelical College of Goianésia, Goianésia, Brazil; 3 University of Brasilia, Brasilia, Federal District Brazil; 4 Federal University of Tocantins, Palmas, Tocantins Brazil; 5 Brazilian Agricultural Research Corporation, CNPAB, Rio de Janeiro, Brazil; 6 Graduate Studies in Natural Resources of the Cerrado, State University of Goiás, Anápolis, Goiás Brazil

**Keywords:** Agroecology, Agroecology

## Abstract

Organic production systems are increasingly gaining market share; however, there are still few studies on their influence on the activity of soil microorganisms in sugarcane. Arbuscular mycorrhizal fungi are extremely sensitive to environmental changes, and their activity can be used as a parameter of comparison and quality between organic and conventional systems. The objective of this work was to evaluate mycorrhizal activity in different varieties of sugarcane under two production systems. This work was carried out in a commercial plantation of the Jalles Machado plant in the municipality of Goianésia in Goiás, Brazil. The values of spore density in the soil, mycorrhizal colonization rate in the roots and easily extractable glomalin were evaluated, and the associated fungal species were identified. There was no effect of sugarcane variety on the number of spores or the glomalin content in the soil. The conventional system presented significantly lower mycorrhizal colonization rates than did the organic system. The varieties cultivated under the conventional planting system showed a greater diversity of arbuscular mycorrhizal fungi, where 12 of the 13 different species of mycorrhizal fungi found in both cultivation systems occurred.

## Introduction

The world interest in reducing dependence on fossil fuels and diversifying the energy matrix to mitigate advances in global warming has attracted attention to biofuels, especially sugarcane ethanol, which has promoted the expansion of sugarcane growing areas^1,2^. Brazil is the world's largest producer of sugarcane, and the area cultivated in the 2019 harvest was 8.48 million hectares, where the state of São Paulo stands out as the largest producer, with 46.29% of the planted area, followed by Goiás, Minas Gerais and Paraná^3^.

The growing demand for organic products has brought a new production perspective to the sugar-alcohol agroindustry, with organic production systems increasing their production area each year in relation to the conventional system. Even with the numerous challenges that organic production on an industrial scale brings, the added value in the final product has encouraged an increasing number of farmers to adopt this system^4,5^.

The main difference between organic and conventional systems is the use of insums and pesticides, which are not allowed in certified organic systems. In the organic production of sugarcane, in place of chemical fertilizers, byproducts of alcohol production are used, such as vinasse and filter cake, compounds that, in addition to acting as fertilizers, contribute to the increase in soil organic matter levels^6^.

The addition of organic compounds in organic systems, as well as the application of phytosanitary chemicals in conventional systems, directly influences the edaphytic microbiota, where arbuscular mycorrhizal fungi (AMF) stand out as plant growth-promoting organisms that affect the health of the associated plants^7,8^.

These fungi, when associated with plants, act as an extension of the roots, searching in soil regions where the plant does not reach water and nutrients, especially phosphorus^9^. These organisms also provide other benefits to the vegetables in which they are associated, such as increased tolerance to water stress and heavy metals, phosphoric solubilization and phytohormone production^10–13^.

These organisms are extremely sensitive to environmental changes, and in this case, they can be used as bioindicators of environmental quality and as a parameter for comparing the quality of organic and conventional sugarcane production systems^14^.

Few studies have evaluated the influence of sugarcane management systems on the community of arbuscular mycorrhizal fungi. Agricultural practices can significantly influence the composition of the AMF community. The objective of this work was to evaluate mycorrhizal activity in different varieties of sugarcane under two production systems.

## Materials and methods

The samples were taken from the commercial crop of the Jalles Machado SA plant in the municipality of Goianésia, Goiás, located at the geographic coordinates 15°12′49.6′′ S 48°59′06.1′′ W in plots with three years of cultivation. The soil of the area is classified as Red Latosol. The climate on site is classified as tropical station (Aw), which is characterized by two well-defined seasons (dry and rainy), as well as the occurrence of drought periods during the rainy season. The results of the soil analysis of the area where the samples were taken are shown in Table 1.

**Table 1:** Soil analysis at a depth of 0–10 cm in a conventional and organic sugarcane production area of Jalles Machado S/A commercial plant.

Production system	ph	P	K	Ca	Mg	H + Al	MO	Sand	Silt	Clay
	Cacl	mg dm^3^	mmol_C_ D^−3^				g kg^−3^	g kg^−1^		
Conventional	5.6	5.68	1.70	48.2	5.0	26.4	11.9	468	86	446
Organic	5.2	3.26	2.72	56.2	6.0	25.6	18.2	232	162	606

The experimental design was completely randomized in a subplot scheme with five replications. The plots were composed of two production systems, conventional and organic; the subplots were the average/late varieties of sugarcane of the third year of cultivation: CTC 4, IACSP 94-2101 and IACSP 95-5000.

The organic cultivation area was managed with the application of 15 Mg ha^−1^ filter cake and 230 m^3^ ha^−1^ vinasse. The conventional cultivation area was treated with 100 Mg ha^−1^ nitrogen and K_2_O and 30 Mg ha^−1^ P_2_O_5_. The filter cake had an average dry matter composition of 1.8% N, 3.4% P, 0.7% K and 99% organic matter.

Three simple samples were randomly collected from plots of 1 hectare at a depth of zero to 20 cm with Dutch Tranto, roots with rhizosphere soil of sugarcane varieties, and for each treatment, three simple samples were collected, forming a compound. The soil of the composite samples was homogenized and stored under refrigeration until spore counting. Root samples were washed and kept in 50% alcohol until evaluation. No type of chemical management is carried out to control invasive plants and insects in organic cultivation areas.

To determine the percentage of colonization, the roots were clarified and cordoned with 0.05% Trypan blue in lactoglycerol^16^, and colonization evaluation was performed under a stereoscopic microscope following the technique of intersection of the quadrants^17^. The spores of AMF were extracted from the soil using 50 cm^3^ of each composite sample by wetting^18^ followed by centrifugation in 50% water and sucrose solution. The spores were separated according to their phenotypic characteristics, such as color, size and shape, to construct different morphotypes under a stereoscopic binocular magnifying glass. The Bradford method was used^19^.

GFE was extracted by mixing 1 g of soil with 8 ml of 20 mM sodium citrate (pH 7.0). Afterwards, the mixture was autoclaved for 30 min at 121 °C. Finally, centrifugation was performed for 20 min at 5000 rpm^19,20^. For the quantification of extractable glomalin from the soil, the Bradford method modified by Wright and Upadhyaya^19^ was used, using bovine sero-albumin as the standard protein and a spectrophotometer at a wavelength of 595 nm.

For the identification of the morphological characteristics of the AMF species, the spores were separated according to their morphotypes and mounted on blades with pure polyvinyl-lactoglycol (PVLG) and PVLG mixed with Melzer (1:1 v/v). The identification of AMF was performed at the Mycorrhizal Fungi Laboratory of Embrapa Agrobiology with the aid of an optical microscope equipped with a micrometric eyepiece. To support the identification work, original articles describing the species were provided on the website of the "International Culture Collection of Arbuscular and Vesicular–Arbuscular Mycorrhizal Fungi"^21^.

Data on the number of spores and mycorrhizal colonization were subjected to statistical analysis using Tukey’s test at 5% probability using Assitat^22^. The graphics were prepared by the GraphPad Prism 8 program^23^. Principal component analyses and canonical correspondence analyses were performed by the Statistical Analysis Program Past 3. × ^24^.

## Results

The analysis of variance (Table 2) showed that there was no statistically significant difference between the varieties for any of the parameters evaluated, and among the cultivation systems, there was variation only for mycorrhizal colonization.

**Table 2:** Analysis of variance of glomalin concentration (mg kg^−1^ soil) easily extracted, mycorrhizal colonization rate (%) and number of spores (N0.50 g^−1^ of soil) in three varieties of sugarcane under conventional and organic cultivation systems.

Source of variation	Test F		
	Glomalin	Colonization	Spores
Variety (F1)	0.0824 ns	2.0274 ns	1.2993 ns
Cultivation system (F2)	2.2381 ns	51.7916**	1.0562 ns
Interaction (F1 × F2)	3.6124 ns	1.6627 ns	1.0854 ns

*ns* not significant (*p* >  = .05).

**significant at the 1% probability level (*p* < 0.01).

**Figure 1:**
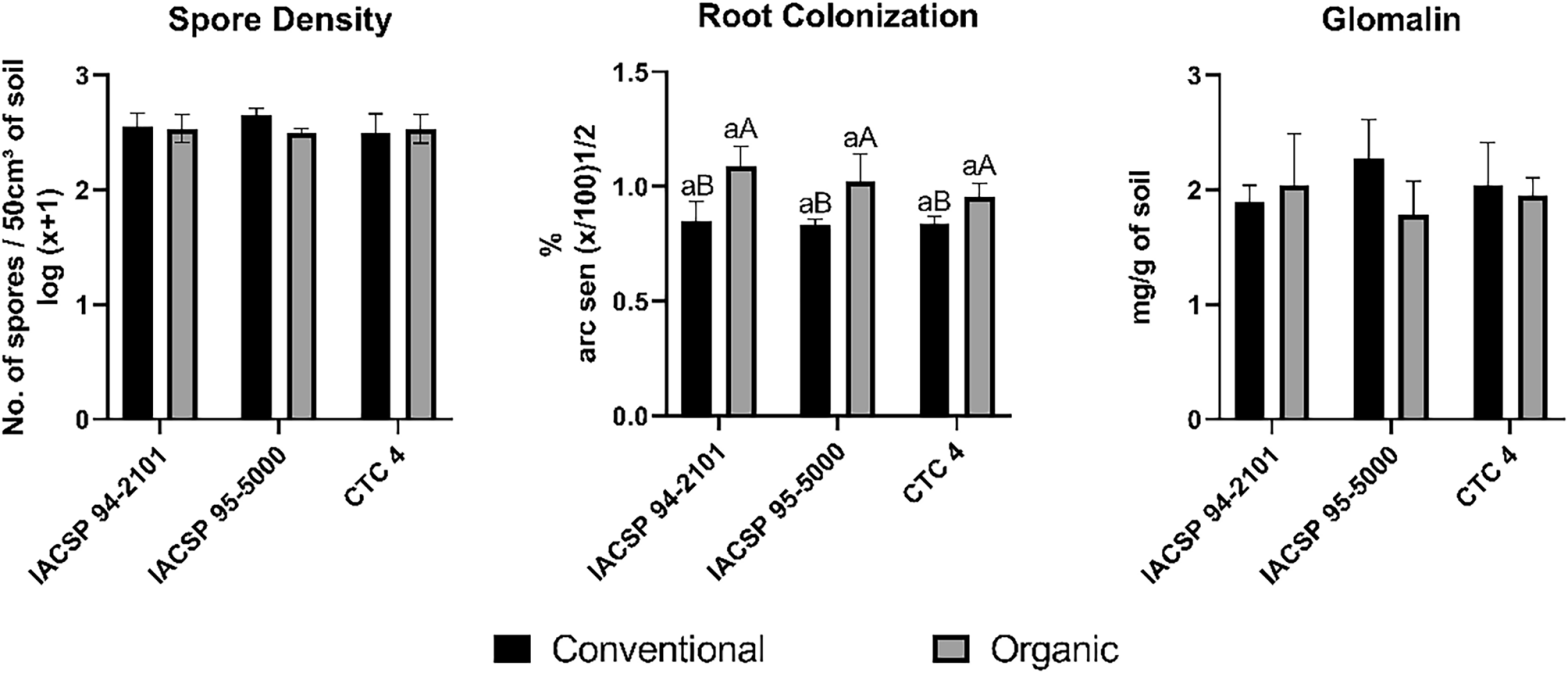
Density of mycorrhizal fungal spores (number of spores/50 cm^3^ of soil), mycorrhizal colonization rate (%) and easily extractable glomalin (mg/g of soil) in three varieties of sugarcane under conventional and organic cultivation systems. Means marked by the uppercase letter correspond to the system, and lowercase letters correspond to the variety according to Tukey’s test (*p* < 0.05). There are error bars in the figure.

There was no significant difference in the spore density of the arbuscular mycorrhizal fungi and glomalin easily extracted in the soil of the sugarcane varieties or in the organic and conventional production systems for all varieties studied. When observing the mycorrhizal colonization rate, a significant difference was verified only between the cultivation systems. All sugarcane varieties grown under the organic production system showed higher rates of mycorrhizal colonization (Fig. 1).

In the identification of associated species found in the rhizosphere of different sugarcane genotypes and cultivation systems, the most frequent individuals belonged to the genera *Gigaspora, Acaulospora, Glomus, Archaeospora* and *Scutellospora* (Table 3).

**Table 3:** Species of arbuscular mycorrhizal fungi found in three varieties of sugarcane cultivated in organic and conventional areas.

Species of arbuscular mycorrhizal fungi	IACSP94-2101	IACSP95-5000	CTC4	IACSP94-2101	IACSP95-5000	CTC4
	Conventional			Organic		
*A. laevis*	−	+	+	+	+	−
*A. scrobiculata*	+	+	+	+	+	+
*A. tuberculata*	+	−	−	−	−	−
*A. spinosa*	+	−	−	+	−	−
*Ar. Leptoticha*	−	−	−	+	−	−
*G. clavisporum*	+	+	+	−	+	+
*C. Lamellosum*	+	−	−	−	−	−
*G. tortuosum*	+	+	+	+	+	+
*G. microaggregatum*	+	−	−	−	−	−
*G. macrocarpum*	+	−	+	−	−	−
*Gigaspora sp*	+	−	−	−	−	−
*S. persica*	+	−	−	−	−	+
*S. pellucida*	−	+	+	−	−	+
Species richness	10	5	6	5	4	5

*C. Claroideoglomus A* = *Acaulospora; Ar* = *Archaeospora; G* = *Glomus; S* = *Scutellospora.*

The species *A. scrobiculata and G. tortuosum* were identified in all genotypes in both systems investigated. The species *A. tuberculata, G. lamellosum, G. microaggregatum* and *G. macrocarpum* were identified only in the areas under the conventional system and *Ar. Leptoticha* was identified only in areas under the organic system.

**Figure 2:**
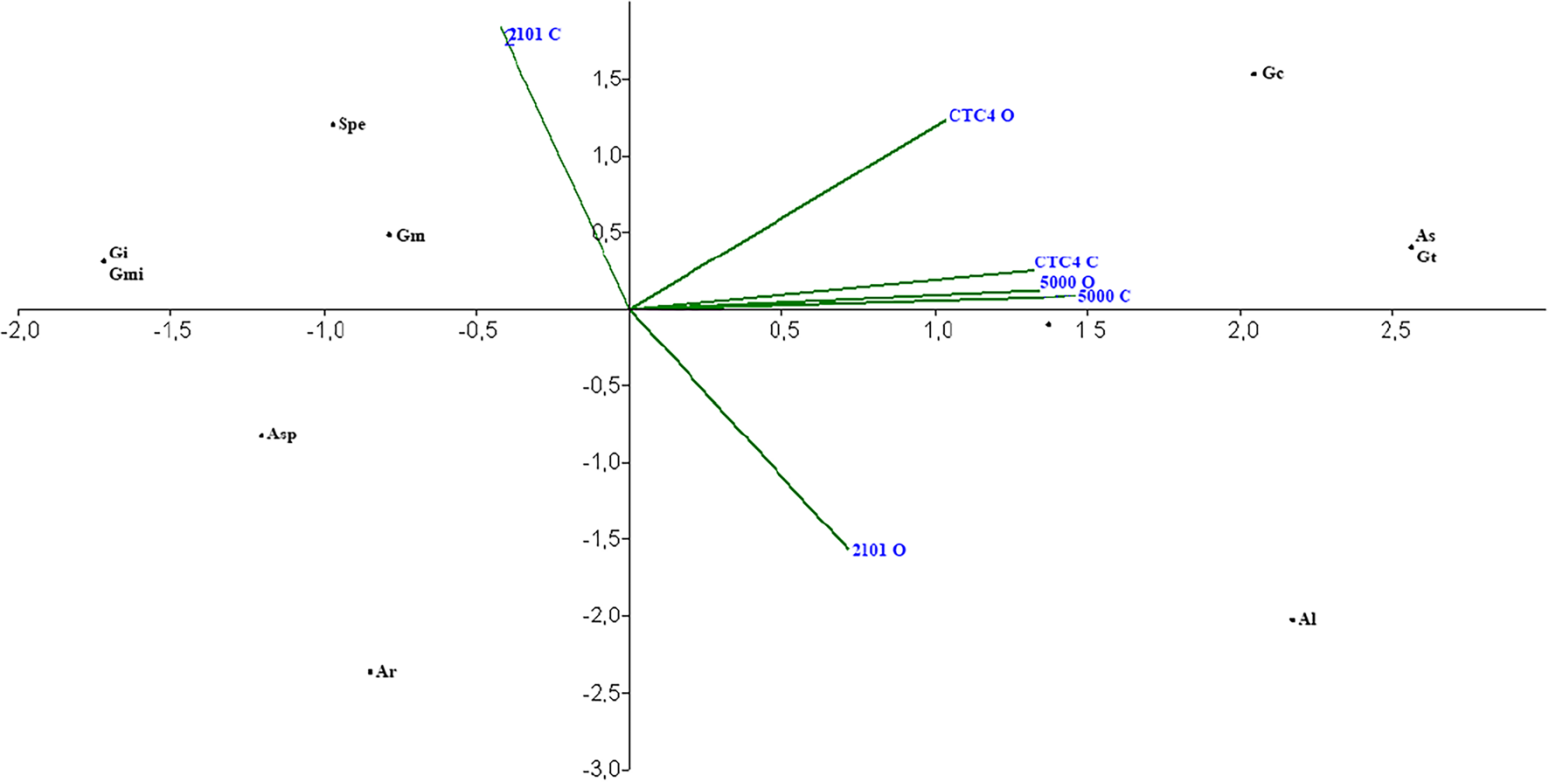
Analysis of the main components of the frequency of AM fungal species in the varieties IACSP94-2101, IACSP95-5000 and CTC4 under organic and conventional systems. (Al: *A. laevis*, Asp: *A. scrobiculata*, As: A. scrobiculata; Air: *Ar. leptoticha,* Ge: *G. etunicatum*, Gm: *G. macrocarpum*, Gmi: *G. microaggregatum*, Gt: *G. tortuosum,* Gi: *Gigaspora* sp., Spe: *Scutellospora pellucida*).

Principal component analysis revealed correlations between the presence of arbuscular mycorrhizal fungal species and the presence of organic and conventional sugarcane varieties (Fig. 2). There is a great distance from the iacsp94-2101 variety when grown in organic and conventional systems. Under the organic production system, *S. persica* and *G. macrocarpum* were more common, while *A. laevis* was more closely related to the conventional cultivation system.

The behaviors of the CTC4 and IACSP95-5000 genotypes were similar to those of the AMF species, regardless of the cultivation system adopted. The species *G. clavisporum, A. scrobiculata, G. tortuosum and S. pellucida* were close to the other varieties in both systems and distanced themselves from the other identified species. The genotype IACSP94-2101 under the conventional system presented proximity to species different from those cultivated under the organic system.

Table 4 presents the productivity data of the three varieties of sugarcane grown in organic and conventional systems where mycorrhizal activity was verified.

**Table 4:** Productivity of three sugarcane varieties in organic and conventional cultivation areas.

Productivity	IACSP94-2101	IACSP95-5000	CTC4	IACSP94-2101	IACSP95-5000	CTC4
	Conventional			Organic		
TSH (Mg ha^−1^)	112.8	94.4	108.7	111.9	149.4	81.4
Brix(°)	20.06	19.8	14.1	19.9	18.1	20.7
Fiber (%)	13.4	14.2	12.2	12.5	12.1	14.7
Pol (%)	14.0	13.5	8.9	14.4	13.1	14.4
TSR (kg/Mg)	141.1	135.8	94.0	144.1	132.3	143.9
Purity (%)	85.0	83.5	74.5	86.2	85.9	85.8
TPHa (Mg ha^−1^)	15.8	12.7	9.6	16.1	19.7	11.7

*TSH* tons of sugarcane per ha, *Brix* degrees brix, *Fiber* (%) bagasse biomass after milling, *Pol* apparent sucrose from sugarcane, *TSR* total sugar recovered, *Purity* (%) purity, *TPHa* Pol ton per hectare.

## Discussion

Mycorrhizal activity in a vegetable can be measured by changes in soil spore density, mycorrhizal colonization rate and easily extractable glomalin^14^. These parameters respond differently to environmental variations and stimuli. The density of spores in the soil varies as the fungi associated with plants perceive environmental stresses, such as a lack of nutrients and water stress, increasing their sporulation in the soil to improve their chances of survival^25^.

The values of the mycorrhizal colonization rate represent the effectiveness of the symbiotic association between plants, and stressful situations, such as the absence of phosphorous, can increase mycorrhizal colonization in the roots^13^. Easily extractable glomalin indicates fungal activity in the soil, which can also be influenced by environmental factors such as temperature, nutrient availability and water availability^26^.

The values of spore density, root colonization rate and easily extractable glomalin did not differ among the sugarcane genotypes studied. Genetic variation among genotypes is not sufficient to reflect differences in the behavior of associated mycorrhizal fungi. Similar behavior was verified by^27^ when studying mycorrhizal activity in sugarcane in rotation with legumes and by Reis et al.^28^ by studying the community of mycorrhizal fungi and nitrogen-fixing bacteria in sugarcane. Datta and Kulkarni^29^ investigated the density of spores in forty sugarcane-producing areas in ten districts of Maharastra, India, and found no significant differences between treatments. Similar^28,29^ behavior was^30^ observed in fourteen sugarcane-producing areas in India.

When investigating the influence of production systems on AMF activity, significant differences were detected only in terms of the mycorrhizal colonization rate (Fig. 1). This behavior can be explained by the lower phosphorous levels found in soils under organic cultivation than in soils under conventional cultivation. These values differ because of the different fertilization systems used in organic areas, which use alcohol residue as a filter and vinasse cakes as fertilizer, and chemical fertilization is used in conventional areas (Table 1).

The scarcity of phosphorous in soils stimulates mycorrhizal colonization in plants, where it acts as an extension of the roots, increasing the root exploration area^12^, and some fungal species also act in the solubilization and availability of phosphorous that is adsorbed to soil colloids. Those from the cerrado are acidic, oxidic and very weathered^31^. These soils have a great capacity for adsorbing phosphorus molecules. Even under conditions of fertilization, plants cultivated in these soils are often scarce^32^.

There are few studies evaluating the influence of organic and conventional systems on the rate of mycorrhizal colonization in sugarcane, but in the present work, the organic system promoted an increase of 22.04% in mycorrhizal colonization. In other crops, under organic and conventional systems, the results are not conclusive. In the onion rhizosphere,^33^ the colonization rate was greater in organic systems (85%) than in conventional systems (72%). In the wheat crop,^34^ mycorrhizal colonization was greater in the organic system than in the conventional system. On the other hand, in vines under conventional and organic cultivation systems during three harvests,^35^ found no differences in the mycorrhizal colonization rate.

Another aspect that may have contributed to a higher rate of mycorrhizal colonization in the organic system is that conservation cultivation systems, such as organic systems, provide a more favorable environment for the exudation of root substances, which ultimately attracts a greater number of rhizospheric microorganisms^36–38^.

Glomalin is a hydrophobic, recalcitrant and thermostable protein that can be produced by soil organisms, especially arbuscular mycorrhizal fungi, and was initially isolated from fungi of the *genus Glomus*^39,40^. Soil characteristics, climatic conditions, land use systems, agricultural management practices, and the presence and type of vegetation, among other factors, influence the amount of glomalin produced by AMF. The production systems, as well as the genotypes investigated, did not impact the differences enough to influence the production of glomalin by fungi (Fig. 1).

Regarding species diversity (Table 3), the composition of the AMF community differed among the studied varieties and management systems. Three species of AMF *belonging to the genera Acaulospora, Archaeospora, Gigaspora, Scutellospora and Glomus were identified in the soil of the production systems and varieties of sugarcane* (Table 3).

The soil cultivated with sugarcane varieties under the conventional planting system showed a greater richness of arbuscular mycorrhizal fungal species. Of the 13 species of mycorrhizal fungi found under the roots of the 3 sugarcane varieties in both cultivation systems, 12 occurred in the conventional system, except *for Ar. Leptoticha.* In the rhizosphere of the same varieties of sugarcane cultivated under the organic system, only 8 of the 13 species of mycorrhizal fungi were found.

The plants that develop under stress conditions are usually associated with arbuscular mycorrhizal fungi, which aid in the absorption of water and nutrients, minimizing adverse conditions^41–43^. In addition to the application of chemical fertilization pesticides, organic and conservation systems are less stressful than systems in which the soil is revolving^44^; therefore, in conventional systems, greater variability of arbuscular mycorrhizal fungal species may occur than in conventional systems^45^.

In general, the variety with the highest diversity of species in the rhizospheric soil was IACSP94-2101 in a conventional planting system, with 10 identified species, which were *A. scrobiculata, A. tuberculata, A. spinosa, G. clavisporum, G. lamellosum, G. tortuosum, G. microaggregatum, G. macrocarpum, S. persica* and the genus *Gigaspora* sp. In the organic system, for this same variety, only five species of arbuscular mycorrhizal fungi were found: *A. laervis, A. scrobiculata, A. sipinosa, Ar. leptoticha* and *G. tortuorum* (Table 3).

Although the production systems are under the same climate conditions and soil type, fertility and organic matter levels are different, which may have influenced the occurrence of different species in the two production systems^45,46^.

When analyzing the frequency of species in conservation systems and areas with soil revolving, Gai et al.^43^, as shown in Fig. 2, they did not find a distance from species considering soil management systems. The species *S. pellucida, G. clavisporum, G. tortuosum, G. macrocarpum, A. scrobiculata and Gigaspora* sp. *were* also found in the wheat, tomato and corn rhizospheres.

Cui et al.^47^ also did not find colonization of Gigaspora or S. gregaria in wheat independent of the soil management system, a behavior similar to that of the varieties analyzed. Moreover, Ferreira et al.^48^ and Higo et al.^49^ identified the presence of *S. pellucida, G. clavisporum, G. tortuosum, G. macrocarpum, A. scrobiculata and Gigaspora* sp.associated with wheat*.*

The production variables can be divided into sugar production, verified in Brix, Pol, TSR, Purity and TPHa, and vegetative growth, verified in TSH and Fiber. These measures directly reflect sugarcane productivity (Table 4). The TSH and fiber variables indicate the biomass production and vegetative growth of the crop. Sugar production and biomass production are normally interconnected; however, physiological changes can influence sugar production without necessarily altering plant biomass production.

This explains why areas that presented relatively low biomass production presented sugar production that was compatible with areas with relatively high biomass production. Physiologically, sugarcane tends to accumulate sugar when subjected to stressful situations^50^. This technique is used as a management practice when herbicide subsoils are applied to plants to reduce stress and stimulate sugar production. Mycorrhizal activity in plants is stimulated when the plant is under stress.

Cultivars produced in the organic system showed greater mycorrhizal activity; however, their productivity was similar to that of cultivars produced in the conventional system. This is probably because in the organic area, due to the absence of fertilizers, the plant was subjected to more stressful situations than in conventional areas, which stimulated AMF activity. Even though the crop is subjected to greater stress in organic areas, fungi may be responsible for maintaining production in these areas.

## Conclusions

There was no effect of sugarcane variety on the number of spores or the glomalin content in the soil. The conventional system presented significantly lower mycorrhizal colonization rates than did the organic system. The varieties cultivated under the conventional planting system showed a greater diversity of arbuscular mycorrhizal fungi, where 12 of the 13 different species of mycorrhizal fungi found in both cultivation systems occurred.

The behavior of the CTC4 and IACSP95-5000 varieties was similar to that of the other AMF species, regardless of the cultivation system adopted. The species *G. clavisporum, A. scrobiculata, G. tortuosum* and *S. pellucida* are close to the other varieties in both systems and are distant from the other identified species. In the variety IACSP94-2101, the frequency of species did not differ between the organic and conventional cultivation systems. The species *G. macrocarpum, G. tortuosum, A. spinosa, G. clavisporum, G. microaggregatum* and *G. lamellosum* are commonly associated with this variety. However, the species *Ar. leptoticha, Gigaspora, S. pellucida* and *A. denticulata*, although present, are not often found to be associated with the variety IACSP94-2101. In the IACSP95-5000 and CTC4 varieties, the organic and conventional systems did not differ in terms of the frequency of AMF species that colonize the rhizosphere.

Although mycorrhizal activity was greater in the organic system, the productivity of the areas was similar. Even though the crop is subjected to greater stress in organic areas, fungi may be responsible for maintaining production in these areas.

## Data Availability

The datasets generated during and/or analyzed during the current study are available from the corresponding author upon reasonable request.
